# 3-Methyl-1-tosyl-1*H*-indole-2-carbaldehyde

**DOI:** 10.1107/S160053681205180X

**Published:** 2013-01-12

**Authors:** Priyamvada Pradeep, Sanaz Khorasani, Charles B. de Koning, Manuel A. Fernandes

**Affiliations:** a Molecular Sciences Institute, School of Chemistry, University of the Witwatersrand, PO Wits 2050, Johannesburg, South Africa

## Abstract

The title indole derivative, C_17_H_15_NO_3_S, crystallizes with two independent mol­ecules in the asymmetric unit. The benzene ring of the tosyl group is almost perpedicular to the indole ring in both mol­ecules, with inter­planar angles of 82.60 (5)° and 81.82 (6)°. The two mol­ecules are, as a consequence, able to form an almost centrosymmetric non-bonded dimer, in which the molecules are linked by pairs of C—H⋯π inter­actions. The crystal structure displays a three-dimensional network of C—H⋯O inter­actions. A π–π inter­action occurs between inversion-related indole rings with a centroid–centroid distance of 3.6774 (16) Å and an inter­planar angle of 1.53 (15)°. This inter­action leads to a stacking of mol­ecules along the *a* axis.

## Related literature

For studies of reactions involving indoles, see: Pathak *et al.* (2006 ▶); Pelly *et al.* (2005 ▶); Sharma *et al.* (2010 ▶). It is inter­esting to note that the reaction used to synthesize this product has been reported to be ineffective when carried out in acetone, see: Kothandaraman *et al.* (2011 ▶).
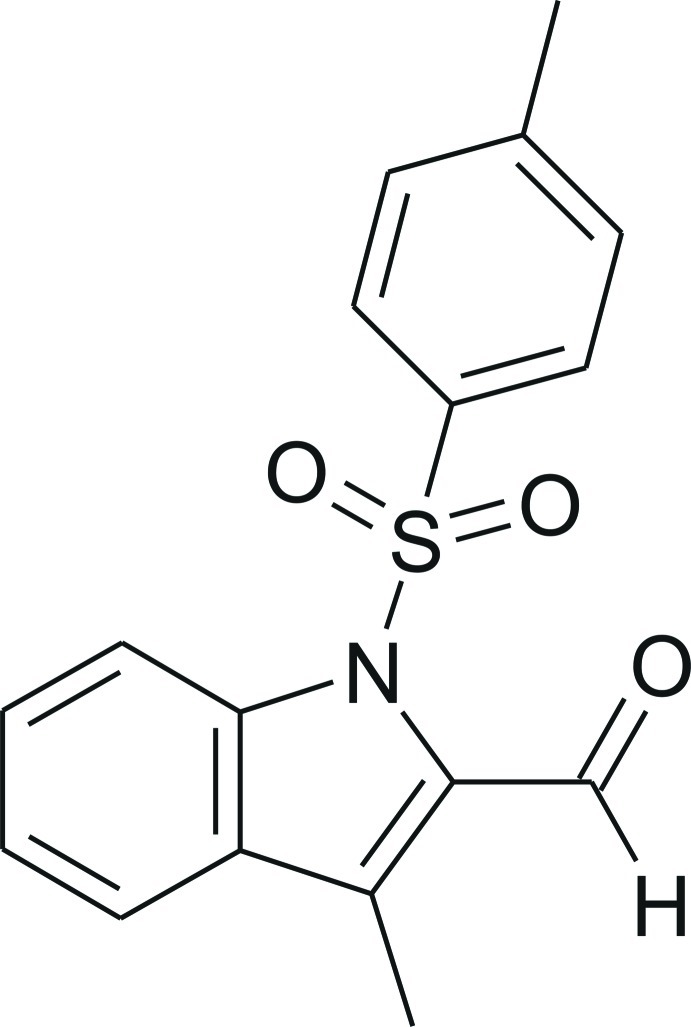


## Experimental

### 

#### Crystal data

C_17_H_15_NO_3_S
*M* *_r_* = 313.36
Triclinic, 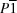

*a* = 8.4276 (2) Å
*b* = 13.0126 (3) Å
*c* = 14.2522 (4) Å
α = 79.968 (2)°
β = 79.794 (2)°
γ = 83.505 (2)°
*V* = 1509.25 (7) Å^3^
*Z* = 4
Mo *K*α radiation
μ = 0.23 mm^−1^
*T* = 173 K
0.28 × 0.25 × 0.05 mm

#### Data collection

Bruker APEX-II CCD diffractometer
16378 measured reflections
5936 independent reflections
3668 reflections with *I* > 2σ(*I*)
*R*_int_ = 0.051

#### Refinement

*R*[*F* ^2^ > 2σ(*F* ^2^)] = 0.049
*wR*(*F* ^2^) = 0.126
*S* = 1.01
5936 reflections
401 parameters
H-atom parameters constrained
Δρ_max_ = 0.31 e Å^−3^
Δρ_min_ = −0.27 e Å^−3^

### 

Data collection: *APEX2* (Bruker, 2005 ▶); cell refinement: *SAINT-NT* (Bruker, 2005 ▶); data reduction: *SAINT-NT*; program(s) used to solve structure: *SHELXS97* (Sheldrick, 2008 ▶); program(s) used to refine structure: *SHELXL97* (Sheldrick, 2008 ▶); molecular graphics: *ORTEP-3 for Windows* (Farrugia, 2012 ▶) and *SCHAKAL-99* (Keller, 1999 ▶); software used to prepare material for publication: *WinGX* (Farrugia, 2012 ▶) and *PLATON* (Spek, 2009) ▶.

## Supplementary Material

Crystal structure: contains datablock(s) global, I. DOI: 10.1107/S160053681205180X/fy2083sup1.cif

Structure factors: contains datablock(s) I. DOI: 10.1107/S160053681205180X/fy2083Isup2.hkl

Additional supplementary materials:  crystallographic information; 3D view; checkCIF report

## Figures and Tables

**Table 1:** Hydrogen-bond geometry (Å, °) *Cg*3, *Cg*4, *Cg*5 and *Cg*6 are the centroids of the C11*B*–C16*B*, C3*B*–C8*B*, C3*A*–C8*A* and C11*A*–C16*A* rings, respectively.

*D*—H⋯*A*	*D*—H	H⋯*A*	*D*⋯*A*	*D*—H⋯*A*
C16*B*—H16*B*⋯O1*B* ^i^	0.95	2.50	3.153 (3)	126
C12*A*—H12*A*⋯O1*A* ^ii^	0.95	2.48	3.218 (3)	134
C16*A*—H16*A*⋯O2*A* ^iii^	0.95	2.52	3.220 (3)	131
C17*B*—H17*F*⋯O2*B* ^iv^	0.98	2.53	3.396 (4)	147
C8*A*—H8*A*⋯*Cg*3	0.95	2.87	3.804 (3)	167
C9*A*—H9*C*⋯*Cg*4	0.98	2.80	3.726 (4)	158
C9*B*—H9*D*⋯*Cg*5	0.98	2.65	3.576 (4)	158
C9*B*—H9*E*⋯*Cg*6	0.98	2.95	3.755 (4)	140

Symmetry codes: (i) 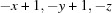; (ii) 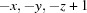; (iii) 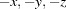; (iv) 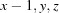.

**Fig. 1.**
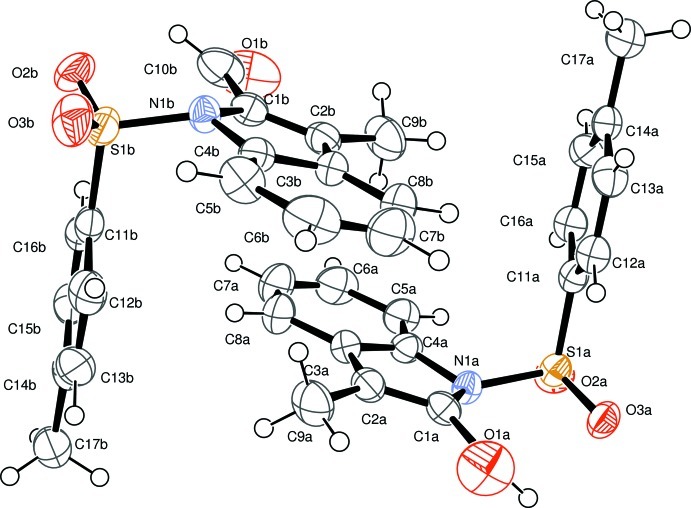
The molecular structure of the title compound, showing the atomic numbering scheme. Displacement ellipsoids are drawn at the 50% probability level.

**Fig. 2.**
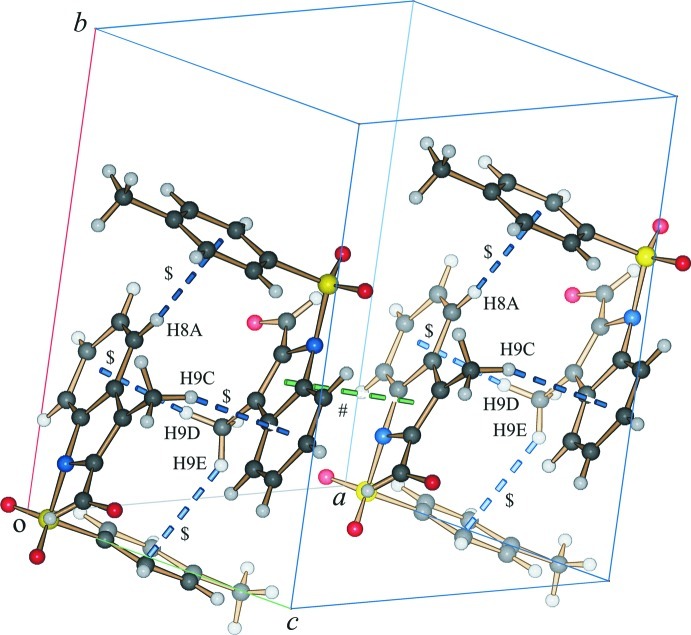
C—H···π and π···π interactions in the title structure. C—H···π and π···π interactions are respectively indicated by dollar ($) or hash (#) signs.

**Fig. 3.**
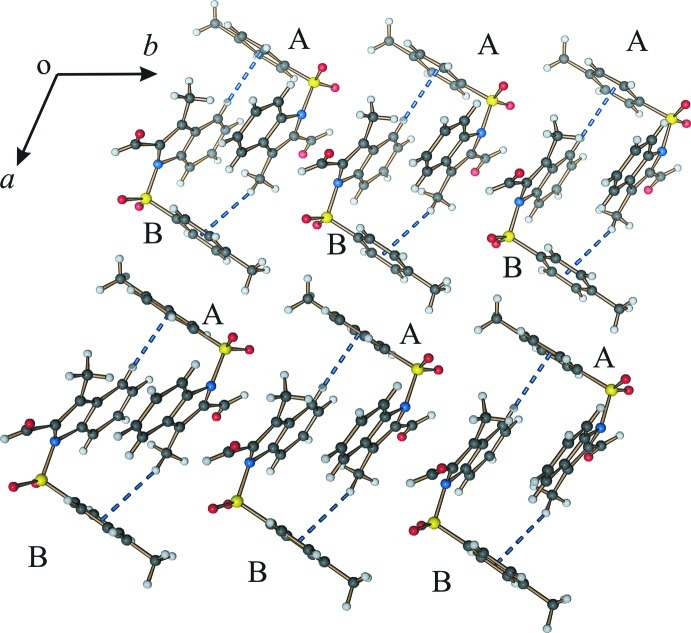
A layer of molecules along the (001) plane, showing the orientation of the A and B molecules with respect to each other. Also shown are the interactions between the molecules which result in molecules A and B forming almost centrosymmetric C—H···π stabilized dimers.

## supplementary crystallographic information

### Comment

Indoles are heterocyclic compounds containing a pyrrole ring fused to a benzene ring at the a,b positions. Indole is an important biological heterocyclic system as it is present, for example, in the amino acid tryptophan. As a consequence, it is a biologically accepted pharmacophore. Derivatives possess a broad spectrum of biological activities (Sharma *et al.*, 2010), for example indomethacin (a non-steroidal anti-inflammatory drug) would fit in this class. The title compound is part of our continuing efforts to synthesize indole-based derivatives (Pathak *et al.*, 2006; Pelly *et al.* 2005)

The title organic compound (Fig. 1) crystallizes in the space group *P*-1 with two independent molecules in the asymmetric unit. The aromatic moieties [indole (C1—C8 and N1) and tosyl group (C11—C17)] in each molecule are orientated with respect to each other at an angle of 82.60 (5)° and 81.82 (6)° in molecules A and B, respectively. The crystal structure contains C—H···O, C—H···π and π···π interactions. The π···π interaction occurs over a *Cg*1···*Cg*2 distance of 3.6774 (16) Å between two indole rings [*Cg*1 = N1A—C1A—C2A—C3A—C4A; *Cg*2 = N1B—C1B—C2B—C3B—C4B] with an interplanar angle of 1.53 (15) ° (Fig. 2). This leads to a stacking of molecules along the *a* axis. The structure contains several C—H···π interactions which are shown in Fig. 2. A layer of A and B molecules along the (001) plane is given in Fig. 3 showing the relative orientation of the A and B molecules in the layer. Geometrical details for the C—H···π and C—H···O interactions are given in Table 1.

### Experimental

The title compound was synthesized by reaction of iodine (942 mg, 3.70 mmol) with *N*-(2-(2-hydroxybut-3-yn-2-yl)phenyl-4-methyl)benzenesulfonamide (584.9 mg,1.854 mmol) in the presence of methanol (20 ml) as a solvent. The resulting mixture was stirred for 6 h at 60°C. The reaction was then quenched by adding a saturated aq. solution of Na_2_S_2_O_3_ and extracted with ethyl acetate (3×20 mL). The combined organics were then washed with aq. NaHCO_3_ and brine, and dried over anhydrous Na_2_SO_4_. After removal of solvent, the left over residue was purified by flash column chromatography, with silica gel using a mixture of hexane and ethyl acetate (20:1) to give 3-methyl-1-tosyl-1*H*-indole-2-carbaldehyde (329 mg, 86%). Single crystals were grown by slow evaporation from dichloromethane.

### Refinement

All H atoms attached to carbon were positioned geometrically, and allowed to ride on their parent atoms, with C—H bond lengths of 0.95 Å (CH) or 0.98 Å (CH_3_), and isotropic displacement parameters set to 1.2 (CH) or 1.5 times (CH_3_) the *U*_eq_ of the parent atom.

### Crystal data

**Table:**

C_17_H_15_NO_3_S	*Z* = 4
*M**_r_* = 313.36	*F*(000) = 656
Triclinic, *P*1	*D*_x_ = 1.379 Mg m^−^^3^
Hall symbol: -P 1	Mo *K*α radiation, λ = 0.71073 Å
*a* = 8.4276 (2) Å	Cell parameters from 2992 reflections
*b* = 13.0126 (3) Å	θ = 2.3–28.0°
*c* = 14.2522 (4) Å	µ = 0.23 mm^−^^1^
α = 79.968 (2)°	*T* = 173 K
β = 79.794 (2)°	Plate, colourless
γ = 83.505 (2)°	0.28 × 0.25 × 0.05 mm
*V* = 1509.25 (7) Å^3^	

### Data collection

**Table:**

Bruker APEX-II CCD diffractometer	3668 reflections with *I* > 2σ(*I*)
Radiation source: fine-focus sealed tube	*R*_int_ = 0.051
Graphite monochromator	θ_max_ = 26.0°, θ_min_ = 1.5°
φ and ω scans	*h* = −10→10
16378 measured reflections	*k* = −16→16
5936 independent reflections	*l* = −17→17

### Refinement

**Table:**

Refinement on *F*^2^	Primary atom site location: structure-invariant direct methods
Least-squares matrix: full	Secondary atom site location: difference Fourier map
*R*[*F*^2^ > 2σ(*F*^2^)] = 0.049	Hydrogen site location: inferred from neighbouring sites
*wR*(*F*^2^) = 0.126	H-atom parameters constrained
*S* = 1.01	*w* = 1/[σ^2^(*F*_o_^2^) + (0.0581*P*)^2^] where *P* = (*F*_o_^2^ + 2*F*_c_^2^)/3
5936 reflections	(Δ/σ)_max_ = 0.028
401 parameters	Δρ_max_ = 0.31 e Å^−^^3^
0 restraints	Δρ_min_ = −0.27 e Å^−^^3^

### Special details

**Table:**

Geometry. All e.s.d.'s (except the e.s.d. in the dihedral angle between two l.s. planes) are estimated using the full covariance matrix. The cell e.s.d.'s are taken into account individually in the estimation of e.s.d.'s in distances, angles and torsion angles; correlations between e.s.d.'s in cell parameters are only used when they are defined by crystal symmetry. An approximate (isotropic) treatment of cell e.s.d.'s is used for estimating e.s.d.'s involving l.s. planes.
Refinement. Refinement of *F*^2^ against ALL reflections. The weighted *R*-factor *wR* and goodness of fit *S* are based on *F*^2^, conventional *R*-factors *R* are based on *F*, with *F* set to zero for negative *F*^2^. The threshold expression of *F*^2^ > σ(*F*^2^) is used only for calculating *R*-factors(gt) *etc*. and is not relevant to the choice of reflections for refinement. *R*-factors based on *F*^2^ are statistically about twice as large as those based on *F*, and *R*- factors based on ALL data will be even larger.

### Fractional atomic coordinates and isotropic or equivalent isotropic displacement parameters (Å^2^)

**Table:**

	*x*	*y*	*z*	*U*_iso_*/*U*_eq_	
C1A	−0.1003 (3)	0.1746 (2)	0.32889 (17)	0.0336 (6)	
C2A	−0.0243 (3)	0.2622 (2)	0.32277 (19)	0.0378 (7)	
C3A	0.0263 (3)	0.2996 (2)	0.22233 (19)	0.0377 (6)	
C4A	−0.0216 (3)	0.2323 (2)	0.16787 (18)	0.0351 (6)	
C5A	0.0136 (3)	0.2468 (2)	0.06829 (19)	0.0440 (7)	
H5A	−0.0172	0.1999	0.0319	0.053*	
C6A	0.0963 (4)	0.3335 (3)	0.0240 (2)	0.0577 (9)	
H6A	0.1225	0.3464	−0.0444	0.069*	
C7A	0.1418 (4)	0.4021 (3)	0.0776 (2)	0.0621 (9)	
H7A	0.1975	0.4610	0.0450	0.075*	
C8A	0.1078 (4)	0.3861 (2)	0.1755 (2)	0.0529 (8)	
H8A	0.1392	0.4332	0.2114	0.063*	
C9A	−0.0073 (4)	0.3179 (2)	0.4035 (2)	0.0558 (8)	
H9A	−0.0690	0.2846	0.4638	0.084*	
H9B	−0.0488	0.3914	0.3894	0.084*	
H9C	0.1071	0.3141	0.4100	0.084*	
C10A	−0.1948 (4)	0.1188 (2)	0.41602 (18)	0.0440 (7)	
H10A	−0.2867	0.0862	0.4094	0.053*	
C11A	0.0869 (3)	−0.0205 (2)	0.19507 (17)	0.0324 (6)	
C12A	0.1566 (3)	−0.0613 (2)	0.27611 (18)	0.0368 (6)	
H12A	0.0971	−0.0580	0.3388	0.044*	
C13A	0.3128 (3)	−0.1065 (2)	0.2650 (2)	0.0426 (7)	
H13A	0.3609	−0.1339	0.3206	0.051*	
C14A	0.4018 (3)	−0.1130 (2)	0.1740 (2)	0.0398 (7)	
C15A	0.3290 (3)	−0.0719 (2)	0.0937 (2)	0.0426 (7)	
H15A	0.3877	−0.0763	0.0309	0.051*	
C16A	0.1733 (3)	−0.0251 (2)	0.10334 (18)	0.0375 (6)	
H16A	0.1256	0.0038	0.0478	0.045*	
C17A	0.5725 (3)	−0.1633 (2)	0.1625 (2)	0.0585 (9)	
H17A	0.6351	−0.1291	0.1030	0.088*	
H17B	0.5712	−0.2378	0.1593	0.088*	
H17C	0.6222	−0.1559	0.2179	0.088*	
O1A	−0.1603 (3)	0.11289 (18)	0.49508 (14)	0.0689 (7)	
O2A	−0.1671 (2)	0.05043 (15)	0.11669 (12)	0.0436 (5)	
O3A	−0.2055 (2)	−0.02049 (14)	0.29029 (12)	0.0422 (5)	
S1A	−0.11298 (8)	0.03517 (5)	0.20793 (4)	0.03321 (18)	
N1A	−0.1070 (2)	0.15447 (16)	0.23392 (13)	0.0316 (5)	
C1B	0.5657 (3)	0.3352 (2)	0.19951 (17)	0.0356 (6)	
C2B	0.4829 (3)	0.2483 (2)	0.22155 (19)	0.0384 (7)	
C3B	0.4422 (3)	0.2264 (2)	0.32444 (19)	0.0375 (6)	
C4B	0.5034 (3)	0.3020 (2)	0.36399 (17)	0.0345 (6)	
C5B	0.4824 (4)	0.3024 (2)	0.46271 (19)	0.0474 (8)	
H5B	0.5227	0.3548	0.4887	0.057*	
C6B	0.4001 (4)	0.2230 (3)	0.5211 (2)	0.0602 (9)	
H6B	0.3836	0.2208	0.5891	0.072*	
C7B	0.3405 (4)	0.1461 (3)	0.4838 (2)	0.0656 (10)	
H7B	0.2845	0.0926	0.5265	0.079*	
C8B	0.3613 (4)	0.1465 (2)	0.3863 (2)	0.0536 (8)	
H8B	0.3215	0.0933	0.3611	0.064*	
C9B	0.4467 (4)	0.1824 (2)	0.1536 (2)	0.0588 (9)	
H9D	0.3486	0.2128	0.1277	0.088*	
H9E	0.4300	0.1114	0.1880	0.088*	
H9F	0.5376	0.1795	0.1005	0.088*	
C10B	0.6480 (4)	0.3793 (2)	0.1047 (2)	0.0568 (9)	
H10B	0.7340	0.4218	0.1014	0.068*	
C11B	0.4026 (3)	0.5558 (2)	0.28498 (18)	0.0350 (6)	
C12B	0.2877 (4)	0.5569 (2)	0.3677 (2)	0.0465 (7)	
H12B	0.3129	0.5245	0.4290	0.056*	
C13B	0.1363 (4)	0.6062 (2)	0.3589 (2)	0.0510 (8)	
H13B	0.0568	0.6070	0.4151	0.061*	
C14B	0.0965 (3)	0.6543 (2)	0.2710 (2)	0.0446 (7)	
C15B	0.2131 (3)	0.6505 (2)	0.1895 (2)	0.0441 (7)	
H15B	0.1875	0.6822	0.1281	0.053*	
C16B	0.3649 (3)	0.6017 (2)	0.19591 (19)	0.0379 (6)	
H16B	0.4434	0.5995	0.1394	0.045*	
C17B	−0.0688 (4)	0.7093 (3)	0.2647 (3)	0.0652 (10)	
H17D	−0.0698	0.7495	0.2000	0.098*	
H17E	−0.0956	0.7568	0.3127	0.098*	
H17F	−0.1488	0.6574	0.2775	0.098*	
O1B	0.6121 (3)	0.3644 (2)	0.03045 (15)	0.0846 (8)	
O2B	0.7045 (2)	0.53596 (15)	0.20847 (15)	0.0543 (6)	
O3B	0.6359 (2)	0.49819 (16)	0.38571 (14)	0.0553 (6)	
S1B	0.59748 (9)	0.49595 (6)	0.29261 (5)	0.0408 (2)	
N1B	0.5867 (2)	0.36954 (16)	0.28676 (14)	0.0337 (5)	

### Atomic displacement parameters (Å^2^)

**Table:**

	*U*^11^	*U*^22^	*U*^33^	*U*^12^	*U*^13^	*U*^23^
C1A	0.0385 (16)	0.0350 (16)	0.0275 (14)	0.0018 (12)	−0.0051 (11)	−0.0089 (11)
C2A	0.0366 (16)	0.0349 (17)	0.0434 (16)	0.0010 (13)	−0.0083 (13)	−0.0109 (12)
C3A	0.0357 (16)	0.0298 (15)	0.0460 (16)	−0.0025 (12)	−0.0032 (13)	−0.0049 (12)
C4A	0.0327 (15)	0.0343 (16)	0.0349 (15)	−0.0017 (12)	−0.0013 (12)	−0.0008 (12)
C5A	0.0482 (18)	0.0459 (19)	0.0333 (15)	−0.0037 (14)	−0.0031 (13)	0.0025 (13)
C6A	0.060 (2)	0.059 (2)	0.0407 (17)	−0.0007 (17)	0.0054 (16)	0.0130 (16)
C7A	0.062 (2)	0.044 (2)	0.071 (2)	−0.0155 (17)	0.0048 (18)	0.0116 (17)
C8A	0.053 (2)	0.0361 (18)	0.066 (2)	−0.0110 (15)	−0.0048 (16)	−0.0005 (15)
C9A	0.062 (2)	0.056 (2)	0.058 (2)	−0.0030 (17)	−0.0125 (16)	−0.0293 (16)
C10A	0.059 (2)	0.0413 (18)	0.0276 (15)	0.0027 (14)	−0.0013 (13)	−0.0040 (12)
C11A	0.0389 (15)	0.0323 (15)	0.0277 (13)	−0.0090 (12)	−0.0024 (11)	−0.0083 (11)
C12A	0.0443 (17)	0.0370 (16)	0.0307 (14)	−0.0077 (13)	−0.0074 (12)	−0.0059 (11)
C13A	0.0497 (18)	0.0375 (17)	0.0435 (17)	−0.0041 (14)	−0.0197 (14)	−0.0018 (13)
C14A	0.0390 (16)	0.0305 (16)	0.0533 (18)	−0.0076 (13)	−0.0069 (14)	−0.0137 (13)
C15A	0.0426 (17)	0.0450 (18)	0.0418 (16)	−0.0056 (14)	−0.0003 (13)	−0.0169 (13)
C16A	0.0448 (17)	0.0401 (17)	0.0308 (14)	−0.0063 (13)	−0.0090 (12)	−0.0094 (12)
C17A	0.0469 (19)	0.048 (2)	0.084 (2)	−0.0030 (16)	−0.0127 (17)	−0.0167 (17)
O1A	0.0877 (18)	0.0860 (18)	0.0295 (12)	−0.0068 (14)	−0.0072 (11)	−0.0022 (11)
O2A	0.0411 (11)	0.0591 (13)	0.0366 (10)	−0.0050 (9)	−0.0145 (9)	−0.0149 (9)
O3A	0.0450 (12)	0.0386 (11)	0.0412 (11)	−0.0151 (9)	0.0032 (9)	−0.0041 (8)
S1A	0.0365 (4)	0.0362 (4)	0.0287 (3)	−0.0086 (3)	−0.0042 (3)	−0.0074 (3)
N1A	0.0366 (13)	0.0321 (13)	0.0260 (11)	−0.0076 (10)	−0.0031 (9)	−0.0036 (9)
C1B	0.0363 (15)	0.0395 (17)	0.0295 (14)	0.0024 (13)	−0.0036 (12)	−0.0069 (11)
C2B	0.0344 (15)	0.0402 (17)	0.0426 (16)	0.0026 (13)	−0.0068 (12)	−0.0154 (13)
C3B	0.0338 (16)	0.0306 (16)	0.0464 (16)	−0.0043 (12)	−0.0017 (12)	−0.0051 (12)
C4B	0.0346 (15)	0.0331 (15)	0.0324 (14)	−0.0002 (12)	−0.0034 (12)	0.0003 (11)
C5B	0.059 (2)	0.0468 (19)	0.0331 (16)	0.0010 (15)	−0.0058 (14)	−0.0032 (13)
C6B	0.067 (2)	0.070 (2)	0.0305 (16)	0.0054 (19)	0.0038 (15)	0.0071 (16)
C7B	0.063 (2)	0.053 (2)	0.065 (2)	−0.0080 (18)	0.0075 (18)	0.0186 (18)
C8B	0.052 (2)	0.0363 (18)	0.068 (2)	−0.0095 (15)	−0.0011 (16)	−0.0013 (15)
C9B	0.066 (2)	0.055 (2)	0.065 (2)	0.0038 (17)	−0.0207 (17)	−0.0315 (17)
C10B	0.059 (2)	0.057 (2)	0.0419 (18)	0.0087 (17)	0.0091 (16)	0.0001 (15)
C11B	0.0393 (16)	0.0286 (15)	0.0396 (15)	−0.0086 (12)	−0.0067 (12)	−0.0084 (11)
C12B	0.059 (2)	0.0482 (19)	0.0365 (16)	−0.0104 (16)	−0.0064 (14)	−0.0157 (13)
C13B	0.049 (2)	0.056 (2)	0.0523 (19)	−0.0076 (16)	0.0029 (15)	−0.0299 (16)
C14B	0.0428 (17)	0.0321 (17)	0.067 (2)	−0.0043 (13)	−0.0127 (15)	−0.0240 (14)
C15B	0.0510 (19)	0.0342 (17)	0.0510 (18)	−0.0056 (14)	−0.0160 (15)	−0.0084 (13)
C16B	0.0416 (17)	0.0337 (16)	0.0388 (15)	−0.0093 (13)	−0.0035 (13)	−0.0063 (12)
C17B	0.049 (2)	0.052 (2)	0.101 (3)	−0.0003 (17)	−0.0153 (19)	−0.0289 (19)
O1B	0.0863 (19)	0.128 (2)	0.0307 (13)	0.0125 (16)	−0.0052 (12)	−0.0067 (13)
O2B	0.0395 (12)	0.0464 (13)	0.0713 (14)	−0.0175 (10)	0.0006 (10)	0.0051 (10)
O3B	0.0653 (14)	0.0514 (14)	0.0607 (13)	−0.0082 (11)	−0.0325 (11)	−0.0154 (10)
S1B	0.0401 (4)	0.0355 (4)	0.0493 (4)	−0.0103 (3)	−0.0117 (3)	−0.0042 (3)
N1B	0.0397 (13)	0.0295 (13)	0.0307 (12)	−0.0058 (10)	−0.0027 (10)	−0.0023 (9)

### Geometric parameters (Å, º)

**Table:**

C1A—C2A	1.353 (3)	C1B—C2B	1.356 (4)
C1A—N1A	1.434 (3)	C1B—N1B	1.437 (3)
C1A—C10A	1.473 (4)	C1B—C10B	1.457 (4)
C2A—C3A	1.434 (4)	C2B—C3B	1.431 (4)
C2A—C9A	1.497 (3)	C2B—C9B	1.490 (3)
C3A—C8A	1.391 (4)	C3B—C8B	1.397 (4)
C3A—C4A	1.400 (3)	C3B—C4B	1.401 (3)
C4A—C5A	1.381 (3)	C4B—C5B	1.388 (3)
C4A—N1A	1.422 (3)	C4B—N1B	1.416 (3)
C5A—C6A	1.390 (4)	C5B—C6B	1.379 (4)
C5A—H5A	0.9500	C5B—H5B	0.9500
C6A—C7A	1.394 (4)	C6B—C7B	1.390 (4)
C6A—H6A	0.9500	C6B—H6B	0.9500
C7A—C8A	1.357 (4)	C7B—C8B	1.368 (4)
C7A—H7A	0.9500	C7B—H7B	0.9500
C8A—H8A	0.9500	C8B—H8B	0.9500
C9A—H9A	0.9800	C9B—H9D	0.9800
C9A—H9B	0.9800	C9B—H9E	0.9800
C9A—H9C	0.9800	C9B—H9F	0.9800
C10A—O1A	1.200 (3)	C10B—O1B	1.204 (4)
C10A—H10A	0.9500	C10B—H10B	0.9500
C11A—C12A	1.385 (3)	C11B—C16B	1.381 (4)
C11A—C16A	1.387 (3)	C11B—C12B	1.388 (4)
C11A—S1A	1.748 (3)	C11B—S1B	1.749 (3)
C12A—C13A	1.373 (4)	C12B—C13B	1.378 (4)
C12A—H12A	0.9500	C12B—H12B	0.9500
C13A—C14A	1.390 (4)	C13B—C14B	1.379 (4)
C13A—H13A	0.9500	C13B—H13B	0.9500
C14A—C15A	1.391 (4)	C14B—C15B	1.386 (4)
C14A—C17A	1.505 (4)	C14B—C17B	1.502 (4)
C15A—C16A	1.377 (4)	C15B—C16B	1.373 (4)
C15A—H15A	0.9500	C15B—H15B	0.9500
C16A—H16A	0.9500	C16B—H16B	0.9500
C17A—H17A	0.9800	C17B—H17D	0.9800
C17A—H17B	0.9800	C17B—H17E	0.9800
C17A—H17C	0.9800	C17B—H17F	0.9800
O2A—S1A	1.4275 (17)	O2B—S1B	1.424 (2)
O3A—S1A	1.4206 (18)	O3B—S1B	1.4262 (19)
S1A—N1A	1.666 (2)	S1B—N1B	1.675 (2)
			
C2A—C1A—N1A	109.9 (2)	C2B—C1B—N1B	109.8 (2)
C2A—C1A—C10A	126.7 (2)	C2B—C1B—C10B	126.9 (3)
N1A—C1A—C10A	122.2 (2)	N1B—C1B—C10B	122.5 (2)
C1A—C2A—C3A	107.5 (2)	C1B—C2B—C3B	107.5 (2)
C1A—C2A—C9A	127.6 (3)	C1B—C2B—C9B	127.6 (3)
C3A—C2A—C9A	124.8 (2)	C3B—C2B—C9B	124.8 (3)
C8A—C3A—C4A	119.6 (3)	C8B—C3B—C4B	119.1 (3)
C8A—C3A—C2A	131.7 (3)	C8B—C3B—C2B	132.3 (3)
C4A—C3A—C2A	108.7 (2)	C4B—C3B—C2B	108.5 (2)
C5A—C4A—C3A	122.2 (2)	C5B—C4B—C3B	122.4 (2)
C5A—C4A—N1A	130.6 (2)	C5B—C4B—N1B	129.9 (2)
C3A—C4A—N1A	107.2 (2)	C3B—C4B—N1B	107.7 (2)
C4A—C5A—C6A	116.6 (3)	C6B—C5B—C4B	116.6 (3)
C4A—C5A—H5A	121.7	C6B—C5B—H5B	121.7
C6A—C5A—H5A	121.7	C4B—C5B—H5B	121.7
C5A—C6A—C7A	121.6 (3)	C5B—C6B—C7B	122.1 (3)
C5A—C6A—H6A	119.2	C5B—C6B—H6B	118.9
C7A—C6A—H6A	119.2	C7B—C6B—H6B	118.9
C8A—C7A—C6A	121.1 (3)	C8B—C7B—C6B	120.9 (3)
C8A—C7A—H7A	119.5	C8B—C7B—H7B	119.6
C6A—C7A—H7A	119.5	C6B—C7B—H7B	119.6
C7A—C8A—C3A	118.9 (3)	C7B—C8B—C3B	118.8 (3)
C7A—C8A—H8A	120.5	C7B—C8B—H8B	120.6
C3A—C8A—H8A	120.5	C3B—C8B—H8B	120.6
C2A—C9A—H9A	109.5	C2B—C9B—H9D	109.5
C2A—C9A—H9B	109.5	C2B—C9B—H9E	109.5
H9A—C9A—H9B	109.5	H9D—C9B—H9E	109.5
C2A—C9A—H9C	109.5	C2B—C9B—H9F	109.5
H9A—C9A—H9C	109.5	H9D—C9B—H9F	109.5
H9B—C9A—H9C	109.5	H9E—C9B—H9F	109.5
O1A—C10A—C1A	122.6 (3)	O1B—C10B—C1B	123.0 (3)
O1A—C10A—H10A	118.7	O1B—C10B—H10B	118.5
C1A—C10A—H10A	118.7	C1B—C10B—H10B	118.5
C12A—C11A—C16A	120.6 (2)	C16B—C11B—C12B	120.6 (3)
C12A—C11A—S1A	120.00 (19)	C16B—C11B—S1B	119.2 (2)
C16A—C11A—S1A	119.4 (2)	C12B—C11B—S1B	120.2 (2)
C13A—C12A—C11A	119.4 (2)	C13B—C12B—C11B	118.5 (3)
C13A—C12A—H12A	120.3	C13B—C12B—H12B	120.8
C11A—C12A—H12A	120.3	C11B—C12B—H12B	120.8
C12A—C13A—C14A	121.3 (2)	C12B—C13B—C14B	122.1 (3)
C12A—C13A—H13A	119.3	C12B—C13B—H13B	119.0
C14A—C13A—H13A	119.3	C14B—C13B—H13B	119.0
C13A—C14A—C15A	118.3 (3)	C13B—C14B—C15B	118.1 (3)
C13A—C14A—C17A	120.9 (3)	C13B—C14B—C17B	120.4 (3)
C15A—C14A—C17A	120.8 (3)	C15B—C14B—C17B	121.4 (3)
C16A—C15A—C14A	121.3 (3)	C16B—C15B—C14B	121.1 (3)
C16A—C15A—H15A	119.4	C16B—C15B—H15B	119.4
C14A—C15A—H15A	119.4	C14B—C15B—H15B	119.4
C15A—C16A—C11A	119.2 (2)	C15B—C16B—C11B	119.6 (3)
C15A—C16A—H16A	120.4	C15B—C16B—H16B	120.2
C11A—C16A—H16A	120.4	C11B—C16B—H16B	120.2
C14A—C17A—H17A	109.5	C14B—C17B—H17D	109.5
C14A—C17A—H17B	109.5	C14B—C17B—H17E	109.5
H17A—C17A—H17B	109.5	H17D—C17B—H17E	109.5
C14A—C17A—H17C	109.5	C14B—C17B—H17F	109.5
H17A—C17A—H17C	109.5	H17D—C17B—H17F	109.5
H17B—C17A—H17C	109.5	H17E—C17B—H17F	109.5
O3A—S1A—O2A	119.72 (11)	O2B—S1B—O3B	119.64 (13)
O3A—S1A—N1A	105.70 (10)	O2B—S1B—N1B	106.31 (11)
O2A—S1A—N1A	106.01 (11)	O3B—S1B—N1B	105.92 (11)
O3A—S1A—C11A	109.47 (12)	O2B—S1B—C11B	108.81 (12)
O2A—S1A—C11A	109.02 (11)	O3B—S1B—C11B	109.75 (13)
N1A—S1A—C11A	106.01 (11)	N1B—S1B—C11B	105.42 (11)
C4A—N1A—C1A	106.58 (19)	C4B—N1B—C1B	106.39 (19)
C4A—N1A—S1A	121.62 (16)	C4B—N1B—S1B	121.72 (17)
C1A—N1A—S1A	123.55 (17)	C1B—N1B—S1B	122.05 (17)
			
N1A—C1A—C2A—C3A	−2.1 (3)	N1B—C1B—C2B—C3B	2.3 (3)
C10A—C1A—C2A—C3A	−169.6 (2)	C10B—C1B—C2B—C3B	172.1 (3)
N1A—C1A—C2A—C9A	172.6 (3)	N1B—C1B—C2B—C9B	−174.6 (3)
C10A—C1A—C2A—C9A	5.2 (5)	C10B—C1B—C2B—C9B	−4.8 (5)
C1A—C2A—C3A—C8A	179.6 (3)	C1B—C2B—C3B—C8B	−178.5 (3)
C9A—C2A—C3A—C8A	4.6 (5)	C9B—C2B—C3B—C8B	−1.5 (5)
C1A—C2A—C3A—C4A	0.2 (3)	C1B—C2B—C3B—C4B	−0.4 (3)
C9A—C2A—C3A—C4A	−174.8 (3)	C9B—C2B—C3B—C4B	176.7 (3)
C8A—C3A—C4A—C5A	2.0 (4)	C8B—C3B—C4B—C5B	−2.0 (4)
C2A—C3A—C4A—C5A	−178.5 (2)	C2B—C3B—C4B—C5B	179.5 (2)
C8A—C3A—C4A—N1A	−177.7 (2)	C8B—C3B—C4B—N1B	176.7 (2)
C2A—C3A—C4A—N1A	1.9 (3)	C2B—C3B—C4B—N1B	−1.7 (3)
C3A—C4A—C5A—C6A	−1.4 (4)	C3B—C4B—C5B—C6B	1.1 (4)
N1A—C4A—C5A—C6A	178.1 (3)	N1B—C4B—C5B—C6B	−177.3 (3)
C4A—C5A—C6A—C7A	0.2 (5)	C4B—C5B—C6B—C7B	−0.1 (5)
C5A—C6A—C7A—C8A	0.5 (5)	C5B—C6B—C7B—C8B	−0.1 (5)
C6A—C7A—C8A—C3A	0.0 (5)	C6B—C7B—C8B—C3B	−0.8 (5)
C4A—C3A—C8A—C7A	−1.2 (4)	C4B—C3B—C8B—C7B	1.8 (4)
C2A—C3A—C8A—C7A	179.4 (3)	C2B—C3B—C8B—C7B	179.8 (3)
C2A—C1A—C10A—O1A	−34.5 (4)	C2B—C1B—C10B—O1B	24.1 (5)
N1A—C1A—C10A—O1A	159.5 (3)	N1B—C1B—C10B—O1B	−167.3 (3)
C16A—C11A—C12A—C13A	0.0 (4)	C16B—C11B—C12B—C13B	−1.0 (4)
S1A—C11A—C12A—C13A	178.8 (2)	S1B—C11B—C12B—C13B	179.1 (2)
C11A—C12A—C13A—C14A	−0.6 (4)	C11B—C12B—C13B—C14B	−0.3 (4)
C12A—C13A—C14A—C15A	0.3 (4)	C12B—C13B—C14B—C15B	1.3 (4)
C12A—C13A—C14A—C17A	−179.6 (2)	C12B—C13B—C14B—C17B	−178.6 (3)
C13A—C14A—C15A—C16A	0.6 (4)	C13B—C14B—C15B—C16B	−1.0 (4)
C17A—C14A—C15A—C16A	−179.6 (2)	C17B—C14B—C15B—C16B	178.9 (2)
C14A—C15A—C16A—C11A	−1.1 (4)	C14B—C15B—C16B—C11B	−0.2 (4)
C12A—C11A—C16A—C15A	0.8 (4)	C12B—C11B—C16B—C15B	1.2 (4)
S1A—C11A—C16A—C15A	−178.0 (2)	S1B—C11B—C16B—C15B	−178.84 (19)
C12A—C11A—S1A—O3A	−35.0 (2)	C16B—C11B—S1B—O2B	20.3 (2)
C16A—C11A—S1A—O3A	143.9 (2)	C12B—C11B—S1B—O2B	−159.7 (2)
C12A—C11A—S1A—O2A	−167.7 (2)	C16B—C11B—S1B—O3B	153.0 (2)
C16A—C11A—S1A—O2A	11.2 (2)	C12B—C11B—S1B—O3B	−27.1 (3)
C12A—C11A—S1A—N1A	78.6 (2)	C16B—C11B—S1B—N1B	−93.4 (2)
C16A—C11A—S1A—N1A	−102.6 (2)	C12B—C11B—S1B—N1B	86.6 (2)
C5A—C4A—N1A—C1A	177.3 (3)	C5B—C4B—N1B—C1B	−178.3 (3)
C3A—C4A—N1A—C1A	−3.0 (3)	C3B—C4B—N1B—C1B	3.1 (3)
C5A—C4A—N1A—S1A	28.0 (4)	C5B—C4B—N1B—S1B	−32.0 (4)
C3A—C4A—N1A—S1A	−152.42 (18)	C3B—C4B—N1B—S1B	149.36 (19)
C2A—C1A—N1A—C4A	3.2 (3)	C2B—C1B—N1B—C4B	−3.4 (3)
C10A—C1A—N1A—C4A	171.4 (2)	C10B—C1B—N1B—C4B	−173.7 (2)
C2A—C1A—N1A—S1A	151.88 (19)	C2B—C1B—N1B—S1B	−149.54 (19)
C10A—C1A—N1A—S1A	−40.0 (3)	C10B—C1B—N1B—S1B	40.1 (3)
O3A—S1A—N1A—C4A	179.08 (18)	O2B—S1B—N1B—C4B	173.42 (18)
O2A—S1A—N1A—C4A	−52.9 (2)	O3B—S1B—N1B—C4B	45.1 (2)
C11A—S1A—N1A—C4A	62.9 (2)	C11B—S1B—N1B—C4B	−71.2 (2)
O3A—S1A—N1A—C1A	34.9 (2)	O2B—S1B—N1B—C1B	−45.5 (2)
O2A—S1A—N1A—C1A	163.00 (19)	O3B—S1B—N1B—C1B	−173.75 (19)
C11A—S1A—N1A—C1A	−81.2 (2)	C11B—S1B—N1B—C1B	69.9 (2)

### Hydrogen-bond geometry (Å, º)

Cg3, Cg4, Cg5 and Cg6 are the centroids of the C11B–C16B, C3B–C8B, C3A–C8A and C11A–C16A rings, respectively.

**Table:**

*D*—H···*A*	*D*—H	H···*A*	*D*···*A*	*D*—H···*A*
C16*B*—H16*B*···O1*B*^i^	0.95	2.50	3.153 (3)	126
C12*A*—H12*A*···O1*A*^ii^	0.95	2.48	3.218 (3)	134
C16*A*—H16*A*···O2*A*^iii^	0.95	2.52	3.220 (3)	131
C17*B*—H17*F*···O2*B*^iv^	0.98	2.53	3.396 (4)	147
C8*A*—H8*A*···*Cg*3	0.95	2.87	3.804 (3)	167
C9*A*—H9*C*···*Cg*4	0.98	2.80	3.726 (4)	158
C9*B*—H9*D*···*Cg*5	0.98	2.65	3.576 (4)	158
C9*B*—H9*E*···*Cg*6	0.98	2.95	3.755 (4)	140

Symmetry codes: (i) −*x*+1, −*y*+1, −*z*; (ii) −*x*, −*y*, −*z*+1; (iii) −*x*, −*y*, −*z*; (iv) *x*−1, *y*, *z*.

## References

[bb1] Bruker (2005). *APEX2* and *SAINT-NT* Bruker AXS Inc., Madison, Wisconsin, USA.

[bb2] Farrugia, L. J. (2012). *J. Appl. Cryst.* **45**, 849–854.

[bb3] Keller, E. (1999). *SCHAKAL99* University of Freiberg, Germany.

[bb4] Kothandaraman, P., Mothe, S. R., Toh, S. S. M. & Chan, P. W. H. (2011). *J. Org. Chem.* **76**, 7633–7640. 10.1021/jo201208e 21744859

[bb5] Pathak, R., Nhlapo, J. M., Govender, S., Michael, J. P., van Otterlo, W. A. L. & de Koning, C. B. (2006). *Tetrahedron*, **62**, 2820–2830.

[bb6] Pelly, P. C., Parkinson, C. J., van Otterlo, W. A. L. & de Koning, C. B. (2005). *J. Org. Chem.* **70**, 10474–10481. 10.1021/jo051826s 16323860

[bb7] Sharma, V., Kumar, P. & Pathak, D. (2010). *J. Heterocycl. Chem.* **47**, 491–502.

[bb8] Sheldrick, G. M. (2008). *Acta Cryst.* A**64**, 112–122. 10.1107/S0108767307043930 18156677

[bb9] Spek, A. L. (2009). *Acta Cryst.* D**65**, 148–155. 10.1107/S090744490804362X PMC2631630 19171970

